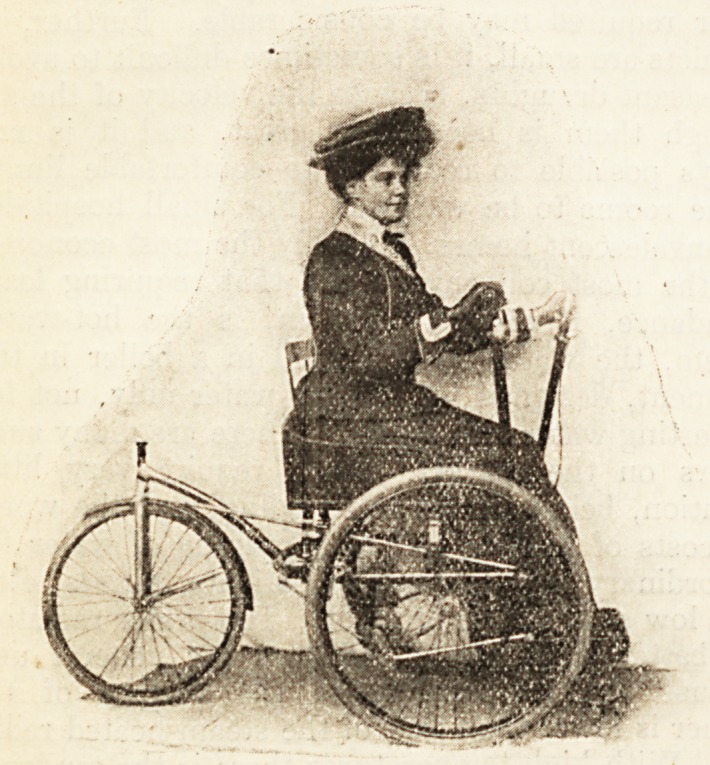# New Appliances and Things Medical

**Published:** 1909-02-13

**Authors:** 


### NEW APPLIANCES AND THINGS MEDICAL.

[We shall be glad to receive at our Office, 28 & 29 Southampton Street, Strand, London, W.C., from the manufacturers, specimens of all new preparations and appliances.]

### AN IMPROVED HAND-PROPELLED CHAIR.

Mr. Jas. P. Witham, of Newport, I.W., writes as follows: As an old patient of the National Hospital for the Paralysed and Epileptic, from which I was discharged as incurable some 13 years ago, I am much interested in the matter of hand-propelled machines for those who cannot walk, and have given much time and trouble to designing a machine for my own use which will obviate the many drawbacks to machines of this class usually supplied. I have been getting about practically all over the Isle of Wight for the last six years in a two-speed and freewheel invalid-chair, but finding the frame around the front (supporting the front wheel) hindered another invalid's getting into my chair; I have arranged it, retaining the excellent steering and propelling action, and putting the steering-wheel at the back. This machine has been so admired by several doctors, by the invalid, and by everyone, in fact, who has seen it, that I send you on a photo in case you might like to illustrate it and give a short description. I imagine many invalids would be pleased to know of such a machine, and I need hardly say I shall be pleased to give anyone full information about it. It has really opened up a new life to me. Of course it can be fitted, like mine, with a freewheel and two-speed gear, and is a very light machine.

### AUTAN.

(F. Bayer and Co., Ebsterfeld, 19, St. Dunstan's Hill, London, E.C.

Autan is a formic aldehyde preparation designed to generate formaldehyde gas upon the addition of water, and thus is especially useful for the disinfection of rooms, cupboards, and wearing apparel. The tin package supplied contains sufficient Autan to disinfect a hundred cubic feet. Ample directions for the disinfection of smaller places are supplied with the package. We regard Autan as a useful and easily applied disinfectant and deodorant.

## Figures and Tables

**Figure f1:**